# Circadian signatures in rat liver: from gene expression to pathways

**DOI:** 10.1186/1471-2105-11-540

**Published:** 2010-11-01

**Authors:** Meric A Ovacik, Siddharth Sukumaran, Richard R Almon, Debra C DuBois, William J Jusko, Ioannis P Androulakis

**Affiliations:** 1 Chemical and Biochemical Engineering Department, Rutgers University Piscataway, NJ 08854, USA; 2 Department of Biological Sciences, State University of New York at Buffalo, Buffalo, NY 14260, USA; 3 Department of Pharmaceutical Sciences, State University of New York at Buffalo, Buffalo, NY 14260, USA; 4 Biomedical Engineering Department, Rutgers University Piscataway, NJ 08854, USA

## Abstract

**Background:**

Circadian rhythms are 24 hour oscillations in many behavioural, physiological, cellular and molecular processes that are controlled by an endogenous clock which is entrained to environmental factors including light, food and stress. Transcriptional analyses of circadian patterns demonstrate that genes showing circadian rhythms are part of a wide variety of biological pathways.

Pathway activity method can identify the significant pattern of the gene expression levels within a pathway. In this method, the overall gene expression levels are translated to a reduced form, pathway activity levels, via singular value decomposition (SVD). A given pathway represented by pathway activity levels can then be as analyzed using the same approaches used for analyzing gene expression levels. We propose to use pathway activity method across time to identify underlying circadian pattern of pathways.

**Results:**

We used synthetic data to demonstrate that pathway activity analysis can evaluate the underlying circadian pattern within a pathway even when circadian patterns cannot be captured by the individual gene expression levels. In addition, we illustrated that pathway activity formulation should be coupled with a significance analysis to distinguish biologically significant information from random deviations. Next, we performed pathway activity level analysis on a rich time series of transcriptional profiling in rat liver. The over-represented five specific patterns of pathway activity levels, which cannot be explained by random event, exhibited circadian rhythms. The identification of the circadian signatures at the pathway level identified 78 pathways related to energy metabolism, amino acid metabolism, lipid metabolism and DNA replication and protein synthesis, which are biologically relevant in rat liver. Further, we observed tight coordination between cholesterol biosynthesis and bile acid biosynthesis as well as between folate biosynthesis, one carbon pool by folate and purine-pyrimidine metabolism. These coupled pathways are parts of a sequential reaction series where the product of one pathway is the substrate of another pathway.

**Conclusions:**

Rather than assessing the importance of a single gene beforehand and map these genes onto pathways, we instead examined the orchestrated change within a pathway. Pathway activity level analysis could reveal the underlying circadian dynamics in the microarray data with an unsupervised approach and biologically relevant results were obtained.

## Background

Circadian rhythms are 24 hour oscillations in many behavioural, physiological, cellular and molecular processes that are controlled by an endogenous clock which is entrained to environmental factors including light, food and stress [1]. These oscillations synchronize biological processes with changes in environmental factors thus allowing the organism to adapt, anticipate, and respond to changes effectively.

Some examples of the biological processes and parameters that show circadian oscillations include body temperature, sleep-wake cycles, endocrine functions, hepatic metabolism and cell cycle progression [2]. Furthermore, disruption of circadian oscillations is linked to many diseases and disorders including cancer, metabolic syndrome, obesity, diabetes, and cardiovascular diseases. In mammals, the central (sometimes referred to as the master) clock is present in the suprachiasmatic nucleus (SCN) in the anterior part of the hypothalamus. Circadian oscillators that are present in other parts of the brain and in other organs are referred to as "peripheral clocks" and are controlled by the central master clock. At the molecular level the clock mechanism involves a transcriptional and post-transcriptional auto-regulatory negative feedback loop consisting of BMAL1 and CLOCK transcription factors which form the positive arm and the PERIOD and CRYPTOCHROME transcription factors which form the negative arm of the feedback loop [3,4]. In addition to these core transcription factors, many other transcription factors which are directly regulated by the core factors including REV-ERBs, RORs and PAR-bZip transcription factors are also involved in the regulation of the circadian expression of the transcriptome which in turn regulates various biological processes [5-7].

Transcriptional analyses of circadian patterns [1,8-10], performed in both drosophila and mammalian systems, demonstrate that genes showing circadian rhythms are part of a wide variety of biological pathways. The expression of several circadian rhythms in a single pathway may ensure a tighter circadian regulation of a pathway or be parts of the circadian clock taking place in other biological functions. The issue of this type of analysis, however, is that moderate but steady changes in the gene expression levels within a pathway could be missed if relatively few individual genes appear significant. Consequently, the identification of biological pathways related to circadian phenomenon could be missed.

We propose to analyze the gene expression data at the pathway level. The starting point of such an analysis is that moderate but steady circadian patterns in the gene expression levels within a pathway could be missed if relatively few individual genes appear circadian. The effectiveness of this approach was illustrated in a study comparing gene expression profiles in muscle of type 2 diabetics (DM2) relative to non-diabetics by [11]. Gene-set enrichment analysis (GSEA) revealed a subset of genes involved in oxidative phosphorylation as being differentially expressed, even though no single gene appeared as differentially expressed between samples. The relationship between oxidative phosphorylation and DM2 is richly supported by the literature [11]. To address the time course gene expression data, Rahnenfuhrer et al. identified the degree of co-expression of genes within a pathway over time [12]. First, the average correlation between gene expression levels within a pathway is computed. Then, the significance of the average correlation of within a pathway is evaluated by a randomization procedure based on the entire microarray. This method, however, can only evaluate whether there is a significant gene expression pattern within a pathway but cannot illustrate the significant pattern itself. Therefore, this method is not able to identify the circadian pattern of a pathway. Alternatively, pathway activity method [13] can identify the significant pattern of the gene expression levels within a pathway. In this method, the overall gene expression levels are translated to a reduced form, pathway activity levels, via singular value decomposition (SVD). A given pathway represented by pathway activity levels can then be as analyzed using the same approaches used for analyzing gene expression levels [13]. Yet, pathway activity method is applied only to evaluate the differentiation between two treatment groups [13,14], i.e. control and treated samples. We propose to use pathway activity method across time to identify underlying circadian pattern of pathways.

Liver is an important organ that is involved in carrying out a wide variety of critical processes including systemic energy regulation processes, metabolism and detoxification of both endogenous and exogenous compounds and hormonal production [9]. Liver is the only tissue that stores glucose in the form of glycogen that can be released in response to glucagon or epinephrine to maintain systemic concentrations [15]. In addition to glucose storage and release, liver can also synthesize glucose de novo through the process of gluconeogenesis. In addition to carbohydrate metabolism, the liver is central to whole body lipid metabolism. About one-half of the cholesterol in the body is produced in the liver, much of which is used for bile acid synthesis [16]. Furthermore, liver is the most important organ that is involved in the metabolism of many drugs and hence contributes to the disposition of these compounds from the body [2]. Proper timing of these processes is of utmost importance for the maintenance of the homeostasis in the system. Previous studies have shown that circadian rhythms are observed at all levels of organization in liver from molecular to the cellular level such as enzyme activity, gene expression, metabolite concentration, DNA synthesis and morphological changes [17]. One of the important levels of organization in the cell is biochemical pathways, which are the ensemble of biochemical reactions to fulfil a particular function. An appreciation of the circadian characteristics of the biological pathways in liver is essential for understanding both the normal physiological and pathophysiological functioning of liver.

In this paper, we used synthetic data to demonstrate that pathway activity analysis can evaluate the underlying circadian pattern within a pathway even when circadian patterns cannot be captured by the individual gene expression levels. In addition, we illustrated that pathway activity formulation should be coupled with a significance analysis to distinguish biologically significant information from random deviations. Next, we performed pathway activity level analysis on a rich time series of transcriptional profiling in rat liver [9]. The over-represented specific patterns of pathway activity levels exhibited circadian rhythms.

## Methods

### Experimental Data

Fifty-four male normal Wistar animals (250-350 g body weight) were housed in a stress free environment with light: dark cycles of 12 hr:12hr. Animals were sacrificed on three successive days at each of 18 selected time points within the 24 hour cycle. The time points were 0.25, 1, 2, 4, 6, 8, 10, 11, 11.75 hr after lights on to capture light period and 12.25, 13, 14, 16, 18, 20, 22, 23, 23.75 h after lights on to capture the dark period. To obtain a clear picture, two 24 hour periods were concatenated to obtain a 48 hour period and are meant only as a visual check that curves do in fact "meet" at the light/dark transitions Our research protocol adheres to the 'Principles of Laboratory Animal Care' (NIH publication 85-23, revised in 1985) and was approved by the University at Buffalo Institutional Animal Care and Use Committee. The details of the experiment can be found in [9]. The data is available under the accession number GSE8988 http://www.ncbi.nlm.nih.gov/geo/.

### Circadian signature of gene expression levels

The circadian pattern of a gene expression is approximated using the sinusoidal model *A *· sin(*B *·*t *+ *C*) [9]. The coefficients are amplitude (A), frequency (B), and phase (C) of the model. The frequency of the sinusoidal model identifies the essence of the circadian behaviour, which is characterized by one full period in 24 hour. The multiplication of total time (t, 24 hr) and frequency (B) should be equal to 2·π in order to characterize one full period (circadian) by the sinusoidal model.

A non-linear curve fitting algorithm is used to define the parameters of the sinusoidal model that would fit best to the gene expression levels over time. The fitted models that have the coefficient B between 0.24 and 0.28 are kept for further analysis to assure the circadian dynamics. Once a model is built for a given gene expression level, the correlation between the data and the model is the criterion to define the circadian signature. Genes are characterized as exhibiting circadian pattern if the correlation between the gene expression and the fitted sinusoidal model is equal or greater than 0.8.

### Pathway Activity Levels

We adapted the pathway activity level formulation to include an additional statistical analysis to evaluate pathway levels [13]. The pathway activity analysis begins with mapping gene expressions of microarray onto pathways. Pathway annotations of gene expressions are retrieved from the publicly available database The Molecular Signatures Database (MSigDB) [18]. Subsequently, gene expression levels within a given pathway are reduced to the pathway activity levels using singular value decomposition (SVD). It is considered that pathway activity levels express the underlying dynamics of a pathway. Next, the significance of the pathway activity levels is evaluated with respect to a randomly permutated microarray data. Then, pathways are filtered out based on the significance analysis.

The matrix Ξ*_P _*(*k,t*) is composed of k genes and t different conditions (correspond to time points and samples) for the gene expression matrix of a given pathway P of size k genes and t samples, and is normalized to have a mean of 0 and a standard deviation of 1. The singular value decomposition (SVD) of Ξ*_P _*(*k,t*)is given as:

(1) $$\Xi_{P}\left(k,t\right)=U_{P}\left(k,k\right)\cdot S_{P}\left(k,t\right)\cdot {V^{\prime }}_{p}\left(t,t\right)\;$$

The columns of the matrix *U_P _*(*k*, *k*)are the orthonormal eigenvectors of Ξ*_P _*(*k,t*). The *S_P _*(*k,t*) is a diagonal matrix containing the associated eigenvalues, and the columns of the matrix ${V^{\prime }}_{p}(t,t)$ are projections of the associated eigenvectors of Ξ*_P _*(*k,t*). As the elements of *S_P _*(*k,t*) are sorted from the highest to the lowest, the first row of ${V^{\prime }}_{p}(t,t)$, represents the most significant correlated gene expression pattern within a pathway across different samples. Pathway activity level, *PAL_P _*(*t*) is defined as the first eigenvector of the ${V^{\prime }}_{p}(t,t)$

(2) $$P\text{A}L_{P}\left(t\right)={V^{\prime }}_{p}\left(t,1\right)\;$$

The first column of *U_P _*(*k*, *k*) is a vector of weights, one weight for each gene within the pathway. The weights can be positive or negative values indicating the direction of the expression levels with respect to the pathway activity levels. A higher absolute weight of a gene specifies a higher contribution to *PAL_P _*(*t*)

The fraction of the overall gene expression (*f_P_*) that is captured by *PAL_P _*(*t*) is:.

(3) $$f_{P}=\frac{S_{P}{\left(1,1\right)}^{2}}{\sum_{g=1}^{t}S_{P}{\left(g,g\right)}^{2}}\;$$

To evaluate whether *PAL_P _*(*t*) can represent significant information of the pathway of interest, referred as the significance analysis of *PAL_P _*(*t*) in this study, we perform an additional analysis. This analysis indicates whether there is significant expression pattern shared by individual genes within a pathway [14]. This is performed by evaluating the significance of the *f_P _*value. First, 10,000 random gene sets of the same size of each pathway are generated from the microarray. Next, the *f_P _*values for the random data sets are evaluated and compared to the actual *f_P _*value. The *p*-value of *f_P _*is computed as the fraction of the *f_P _*of the randomly generated matrices that exceeded the actual *f_P _*. If the *f_P _*of the randomly generated matrices exceeds the actual *f_P _*by more than 5%, then the actual *f_P _*is attributed to a random variation in the microarray data (*p*-value < 0.05). Finally, the pathways are filtered based on the associated *p-value *of their *f_P _*value.

Subsequently, *PAL_P _*(*t*) (Eq. (2)) is applied to describe the pathway activity levels over time. Each entry of *PAL_P _*(*t*) represents the pathway activity level of corresponding experimental condition (Ξ*_P _*(*k,t*)includes replicate measurements at each time point). However, *PAL_P _*(*t*) do not indicate any up-or down-regulation in pathway behaviour, instead *PAL_P _*(*t*) evaluates the relative change across different experimental conditions. The sign *PAL_P _*(*t*) can be chosen based on the pattern the genes that have the highest contribution to *PAL_P _*(*t*) (*PAL_P _*(*t*)≡-*PAL_P _*(*t*)) [13].

### Clustering Analysis of Pathway Activity Levels

To cluster the statistically significant pathway activity levels, we applied an unsupervised clustering approach proposed by Nguyen et al. [19]. This approach was applied to detect the significant clusters of co-expressed genes. In this study, we use pathway activity levels instead of gene expression levels.

First, ANOVA is used as a part of the clustering algorithm of the pathway activity levels, where three replicates of each measurement are averaged [20]. Therefore, we applied ANOVA (p-value < 0.01) to remove the pathway activity levels that are not statistically changing across time points prior to the clustering calculation. ANOVA analysis ensures that the observed changes in pathway activity levels occur over time. Following, repeated measurements are averaged for clustering [20]. Subsequently, the optimum number of clusters are decided after considering several clustering methods (hclust, diana, kmeans, pam, som, mclust), metrics (Euclidian, Pearson correlation, and Manhattan) and an agreement matrix that quantifies the frequency which two pathways belong to the same cluster based on the pathway activity levels. Then a subset of pathways is selected to ensure that no pathway is present with an ambiguous cluster assignment with any other pathway in the analysis with a confidence level *δ*. The *δ *is the threshold to say whether the agreement level of two pathways belong to one (*δ*). or two clusters (1 *-δ*) is consistent or not. The last step is dividing the selected subset into a number of patterns based on the agreement matrix. The details of the algorithm can be found in [19]. In this analysis we use *δ *= 0.65.

### Synthetic Data

A hypothetical pathway that consists of 45 gene expressions across T = 54 samples (3 replicates at 18 time points) is constructed following previously described methods. The gene expression values within the synthetic pathway, gi, are generated based on a widely accepted model of periodic gene expression

(4) $$g_{i}=\beta \cdot \cos(\omega \cdot t+\varphi )+\varepsilon_{t}\;$$

Where *β *is a positive constant, *ω∈*(0, *π*), *φ *uniformly distributed in (*-π, π*] where *ε_t _*is a sequence of uncorrelated random variables with mean 0 and variance *σ*^2 ^, independent of *φ*. We assume *φ *= 0 for all simulated profiles. In order to simulate different signal to noise ratios we also assume the amplitude for baseline variation constant, but add different noise component *ε *for individual profiles. The *ε *value for each fraction was taken as a random number *ε_t _∈*[0,50·*i*], *i *= 0,1,2,...100. When the noise level, i, is zero, all 45 genes have the same circadian pattern. As we increase the noise level, the profiles of the individual gene expressions deviate from the circadian pattern and converge to random variation.

To quantify the effect of the noise level on the individual genes within the synthetic pathway, 1000 replicates of the synthetic pathway are generated at different noise levels. For each generated replicate, the fraction of the circadian genes within the synthetic pathway is evaluated and then compared to a given percentage value, i.e. 50%. If the actual the fraction of the circadian genes within the synthetic pathway is smaller than the 0.5, the event that 50% of the genes within the synthetic pathway are circadian is attributed to a random variable. The ratio of the total number of the event that 50% of the genes within the synthetic pathway are circadian to 1000 identifies the p-value. In addition to p-value for the event that 50% of the genes within the synthetic pathway are circadian, p-values for the event that 10% and 90% of the genes within the synthetic pathway are circadian at different noise level.

We evaluate the *PAL_P _*(*t*) of the synthetic data as the noise level is increased and a non-linear curve fitting algorithm is used to define the parameters of the sinusoidal model that would fit best to the pathway activity levels over time. The procedure for the determination of circadian pattern of pathway activity levels is similar to the determination of circadian pattern of gene expression levels. The synthetic pathway is identified as exhibiting a circadian pattern if the correlation between *PAL_P _*(*t*) and the fitted sinusoidal model is equal to or greater than 0.8.

## Results

### Synthetic Data

**Figure 1:**
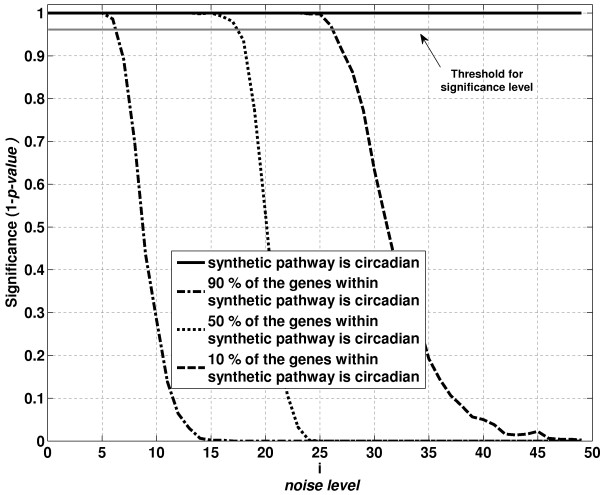
**Effect of noise level on the circadian dynamics of the synthetic pathway**. As the noise level is increased, the significance (1-p-value) of the event that synthetic pathway is circadian and the events that 10, 50 and 90% of the genes within the synthetic pathway are circadian are illustrated. The calculations of the p-values are explained in the methods section.

To test the hypothesis that pathway activity analysis can identify changes that emerge at the pathway level that cannot be identified at the individual gene expression level, a synthetic pathway consisting of 45 genes was constructed and data representative of circadian pattern is generated at different noise levels. Subsequently we compared the significance of the event when 90, 50 and 10% of the genes within the synthetic pathway are circadian. These results are compared with the significance of the synthetic pathway showing circadian pattern in its pathway activity level in Figure 1. For either method, a significance value close to unity indicates that the event is highly likely. A typical threshold used to consider the significance of an event is 0.95. The purpose of this analysis is to evaluate the effect of noise level on the number of genes showing circadian pattern within the pathway.

From Figure 1, we observe that at low noise levels (0 < i < 6) we are confident that at least 90% of the genes within the synthetic pathway are circadian. However, the confidence level of detecting 90% of the genes is circadian decreases sharply as we increase the noise level. At this noise level, the underlying circadian pattern can be identified via both evaluating the circadian genes and pathway activity levels. At a noise level of 17, we can confidently conclude that only 50% of the genes are circadian. At higher noise levels, i.e. i = 30, we cannot even conclude that 10% of the genes are circadian (p-value > 0.05). Thus gene expression alone will not be able to provide information about the significant circadian pattern at this noise level. However, pathway activity analysis predicts with high confidence level (p-value < 0.0001) that there is an underlying circadian pattern within the synthetic pathway at this noise level (i = 30). Therefore, pathway activity levels are more robust than the gene expression levels in identifying underlying expression pattern within a pathway.

**Figure 2:**
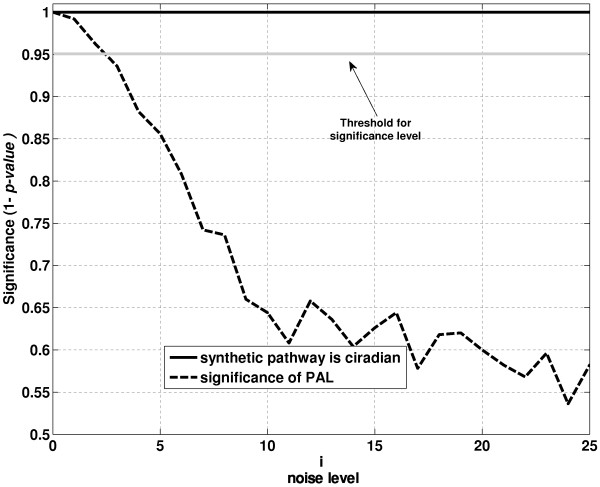
**Effect of noise level on the significance of PAL**. As the noise level is increased, the significance (1-p-value) of the event that synthetic pathway is circadian and the significance of PAL are illustrated.

Nevertheless, a critical issue arises when we consider whether the variation captured by *PAL_P _*(*t*) can represent the overall gene expression within a pathway. While we can be confident that a circadian pattern does exist, we cannot be confident that this pattern is real or due to random variations. To address this issue of random noise in the data vs. real gene expression changes, we evaluated the significance of the *PAL_P _*(*t*) (presented in Figure 2 at different noise levels). Even though *PAL_P _*(*t*) might predict confidently a circadian pattern, that event could be the results of random variability in the data, as quantified by the significance of *PAL_P _*(*t*). For example, at *α *= 10, the significance of the synthetic pathway being circadian is high; however, the significance of *PAL_P _*(*t*) is considerably lower. This result indicates that the observed pattern cannot be solely attributed to the underlying structure of the data. Therefore, determining significance level *PAL_P _*(*t*) is necessary for a reliable representation of circadian pathways.

### Circadian Signatures of Pathways in Rat Liver

We analyzed a rich time series of transcriptional profiling in rat liver where the rats were maintained in 12:12 hours light/dark cycle and exposed to the least possible environmental disturbances to minimize stress. We evaluated pathway activity level analysis on the microarray data and following applied a clustering analysis of the pathway activity levels.

**Figure 3:**
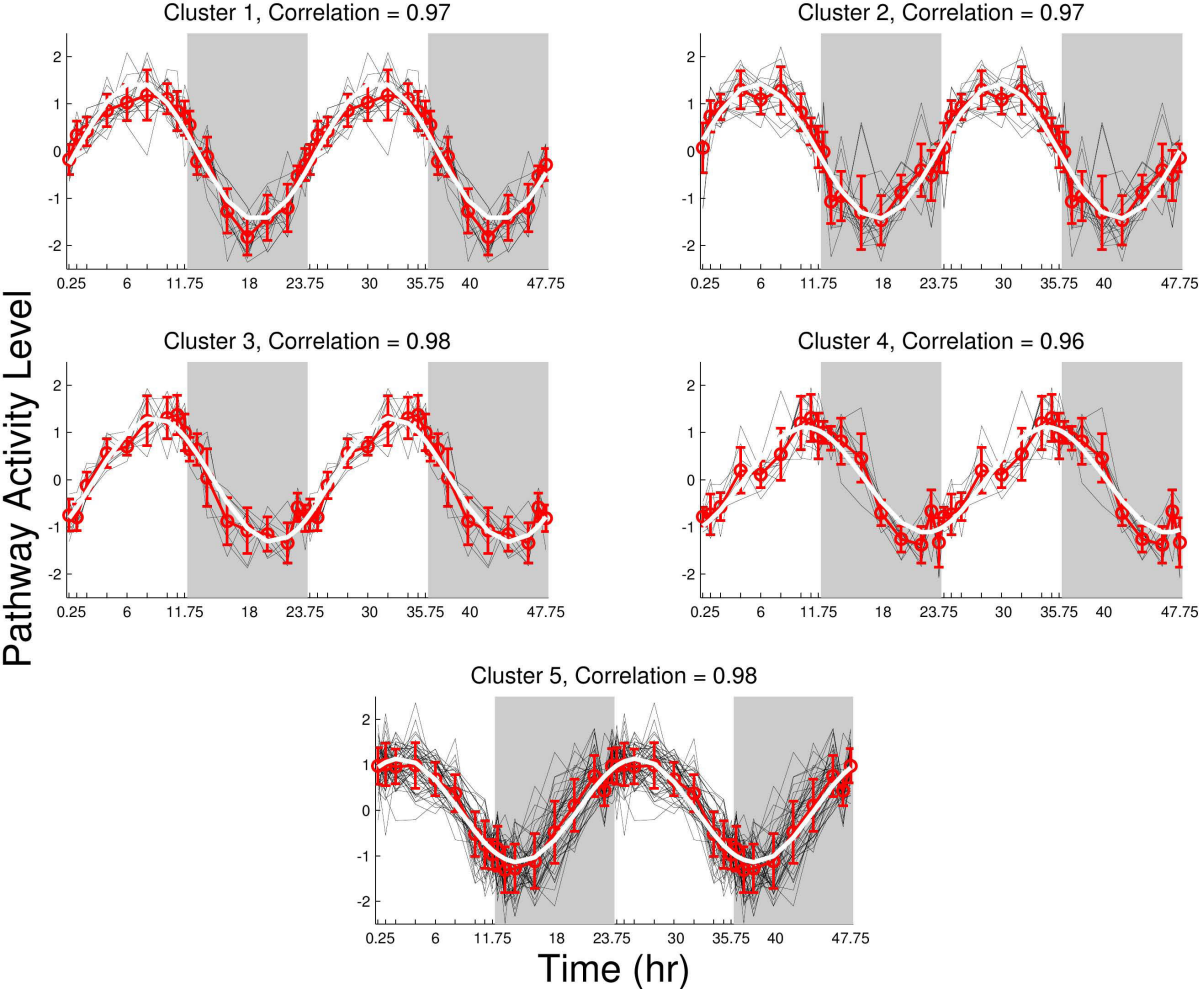
**The five significant clusters identified by a consensus clustering analysis **[19]**using *δ *= 0**.65. The pathway activity level (PAL) of pathways represents the presented curves and the exact reverse curves; PAL = (-) PAL. The signs of PAL are chosen so that PAL has the similar patterns for a better representation and clustering purposes. The centroids of each cluster is shown with the red error bars, the fitted sinusoidal model to the centroids of each cluster is depicted in white.

**Figure 4:**
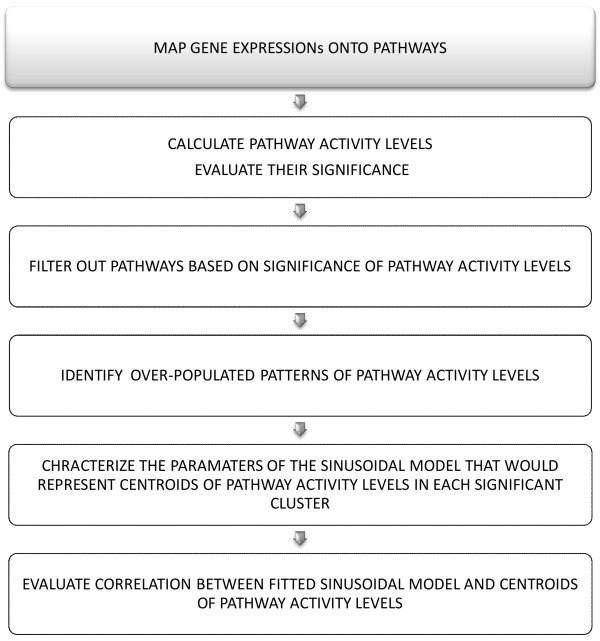
**The outline for clustering analysis of pathway activity levels**. Pathway activity analysis begins with mapping gene expression onto known pre-defined groups of genes, pathways. Subsequently, the pathway activity levels are calculated using SVD and the significance of pathway activity levels are evaluated. Pathways are filtered based on the significance of the PALs. Following, the over-populated patterns are identified by using a consensus clustering approach proposed in [19]. Then, the parameters of the sinusoidal model *A *· sin(*B *· *t *+ *C*) that would best fit the centroids of the pathway activity levels (in each clusters) are characterized. Finally, the correlation between fitted sinusoidal model and the centroids of the pathway activity levels in each cluster is evaluated.

As a result of the significance analysis *f _P _*486 of the 638 defined pathways in MSigDB are considered for further analysis. Having eliminated the pathway activity levels that do not exhibit a significant change over time (ANOVA, p-value < 0.01), the clustering analysis yielded five significant patterns of pathway activity levels (Figure 3). We follow an unsupervised approach and identify the emergent pathway activity level patterns that appeared to have sinusoidal circadian patterns. The significant clusters represent the most populated pathway activity levels patterns within the data, whereas the rest of the data can be associated with random deviations. To quantify the characteristics of the circadian patterns, we perform the approximation of the centroid of each cluster to a sinusoidal function. The correlation between the centroid of each cluster and the associated fitted sinusoidal model exhibit high correlation (correlation = > 0.96, given on top of each graph in Figure 3). The outline of this analysis is depicted in Figure 4.

The peak and nadir points are referred as the turning points. Cluster 1, Cluster 2 have their turning points around the middle of the light period (~6th-8th hours of the 24 hour cycle) and around the middle of the dark period (~18^th ^and 20th hours 24 hour cycle). Cluster3, Cluster 4 and Cluster 5 have their turning points around the transition between the light and the dark period (~10th-13th hours of the 24 hour cycle) and their the turning points around the beginning of the light period and at the end of the dark period (~1^st ^-2^nd ^hours and ~20^th ^and 22^nd ^of the 24 hour cycle).

**Figure 5:**
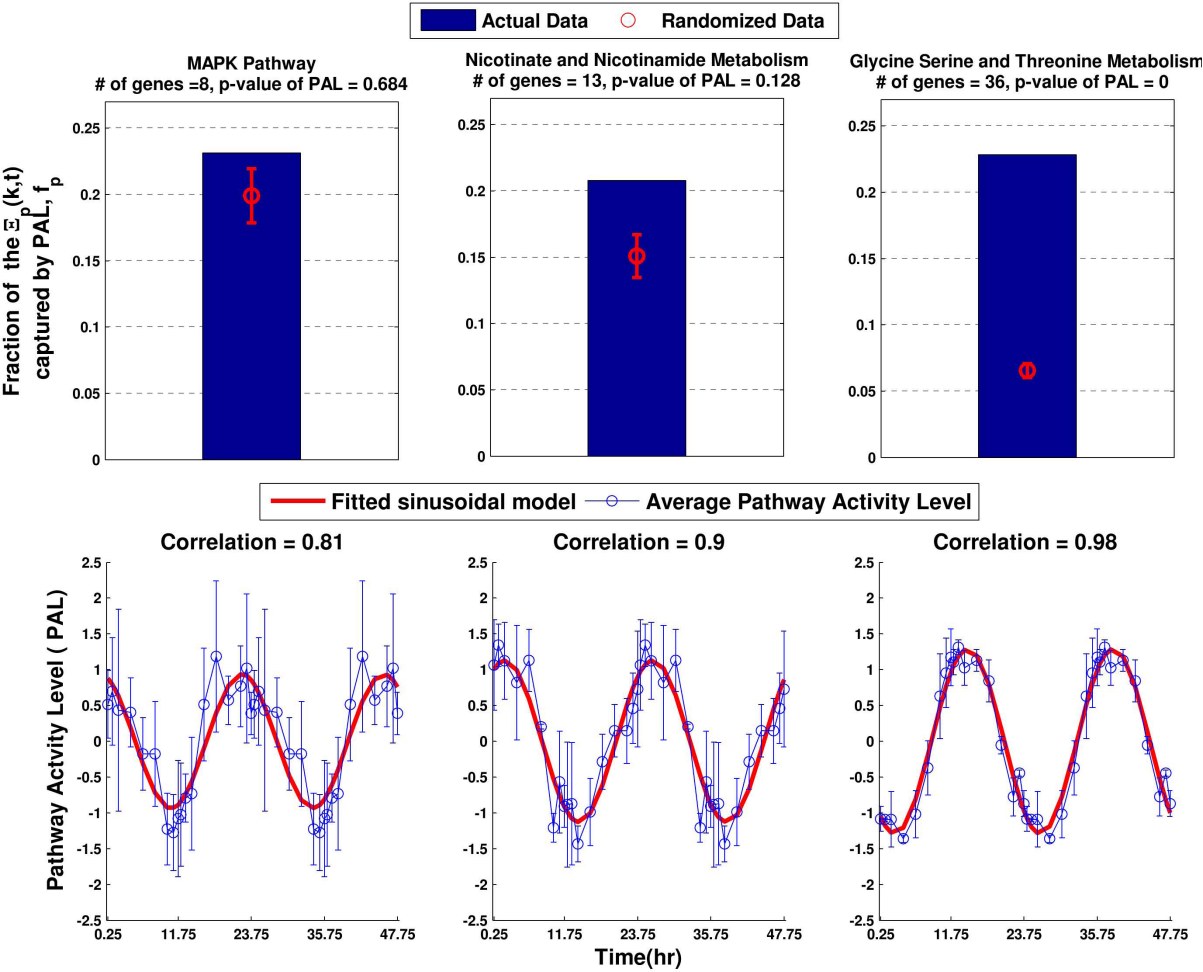
**Pathway activity levels for select pathways**. A) The comparison of the f_p _to the permutated f_p _for MAPK Pathway, nicotinate and nicotinamide metabolism and glycine, serine and threonine metabolism pathway. The mean and the standard deviation interval of permutated *f_p _*is given. The same value of *f_p _*can be obtained by randomly permutated data in MAPK Pathway and nicotinate and nicotinamide metabolism, whereas the *f_p _*captured by randomly permutated data is much lower compared to *f_p _*in glycine, serine and threonine metabolism pathway B) Pathway activity levels and fitted sinusoidal models for the pathways. The mean and the standard deviation interval of the pathway activity levels are given. The correlation between pathways activity level and fitted sinusoidal model is presented for each pathway on top of each graph.

Evaluating pathway activity levels resulted cases where two pathways have similar fraction of overall gene expression captured by *PAL_P _*(*t*), *f_P _*values, however the associated p-values, vary significantly. In example, *f_P _*MAPK Pathway, Nicotinate and nicotinamide metabolism and glycine, serine and threonine metabolism pathway are 0.23, 0.21 and 0.22 respectively (top panel of Figure 5). On the other hand, their associated p-values are rather different; 0.66, 0.12 and 0, respectively (top panel of Figure 5). Depending on the size of the pathways, which is number of the genes within a pathway, *f_P _*value can be obtained from random variations. Therefore, *f_P _*value itself is not an objective feature to identify whether the information captured overall gene expression by *PAL_P _*(*t*)is significant. The significance analysis of *PAL_P _*(*t*) enables us to filter out pathways that exhibit circadian rhythms by chance. For example, MAPK pathway and Nicotinate and nicotinamide metabolism may be identified as exhibiting circadian pattern without the significance analysis of *PAL_P _*(*t*) because *PAL_P _*(*t*) of MAPK Pathway and Nicotinate and nicotinamide metabolism exhibit high correlation with the fitted sinusoidal model (bottom left and bottom middle panels in Figure 5).

**Figure 6:**
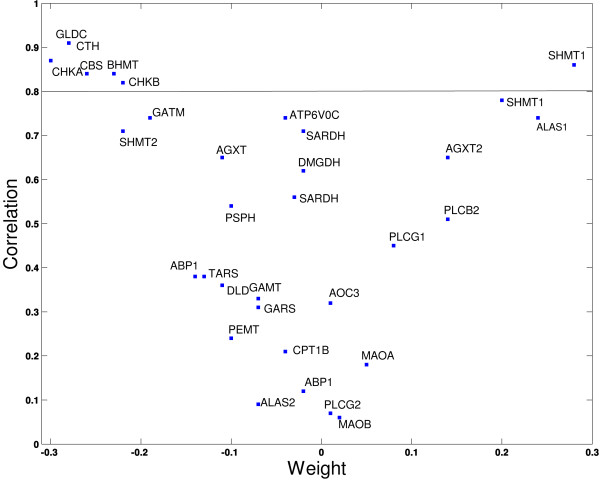
**The relationships between weight and the correlation of the genes within glycine, serine and threonine metabolism**. The correlation is between gene expressions and the fitted sinusoidal models and is set to identify circadian genes. The threshold for circadian genes is correlation > 0.8. The weights are evaluated from the SVD analysis. The absolute value of the weights represents the contribution of the individual genes to the pathway activity level. The genes that have higher correlation values have relatively higher absolute weights.

Glycine, serine and threonine metabolism exhibit both significant *PAL_P _*(*t*) and high correlation with the fitted sinusoidal model (top right and bottom right panels in Figure 5). To study the effect of individual gene expression on the pathway activity level, we depict the relationship between the weights and the correlation of the individual genes (the correlation between gene expression levels and the fitted sinusoidal model that represent the circadian pattern) in glycine, serine and threonine metabolism pathway Figure 6. The weight of a gene characterizes its contribution to the pathway activity level compared to the rest of the genes in the pathway.

**Figure 7:**
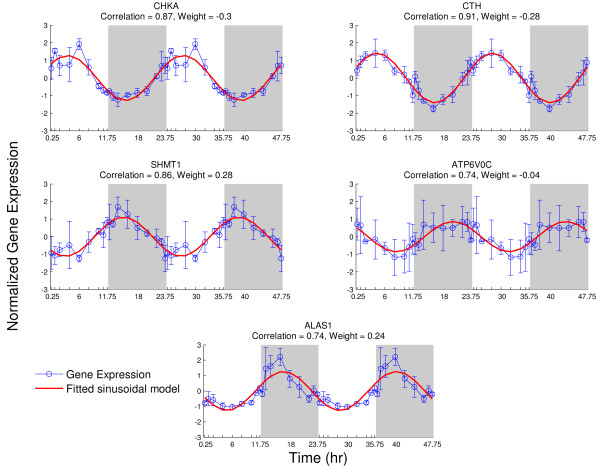
**Selected gene expressions within glycine, serine and threonine metabolism**. The correlation between the gene expression levels and the fitted sinusoidal models and the weights, which are evaluated via SVD analysis, of the genes are given on top of each graph. The signs (positive and/or negative) of weights indicate opposite direction in the gene expression.

It can be seen from Figure 6, that Gldc, Cth, Chka, Chkb, Cbs, Bhmt and Shtm1 exhibit circadian patterns (correlation > 0.8) and also their weights are among the highest (weight > | -0.25|). In addition, the genes, which correlation is slightly under the threshold (correlation ~> 0.7) such as Gatm, Shtm2 and Alas1, have comparably higher absolute weights (weight ~> | -0.25|). The positive and negative values of weights indicate the direction of the gene expression when compared to the pathway activity level. In example, the genes that have negative weights have their peak in the early light period and their nadir in the early dark period (e.g. Chka, Cth), whereas the genes that have positive values have their nadir in the early light period and peak in the early dark period (e.g. Shmt1) (Figure 7.). The pathway activity levels of glycine, serine and threonine metabolism (bottom right panel in Figure 5) follow the genes that have the positive weight value (e.g. Chka, Cth) and have its turning point in the early light period. The sign (positive or negative) of the weights can be chosen to represent pathway activity level as pathway activity levels indicate the overall orchestrated significant change in the gene expression within a pathway. Furthermore, we observe that there are genes, which correlation is slightly under the threshold (correlation ~> 0.7) but they have low absolute weights (weight ~< 0) such as Atp6voc and Sardh. The expression pattern of these genes, (as an example we depicted the expression pattern of Atp6voc in Figure 7) does not coincide with the rest of the genes that have higher absolute weights, therefore do not contribute to the pathway activity level as much and has low weights.

By applying SVD, a number of possible correlated variables (gene expressions) are mapped onto a smaller number of uncorrelated variables (the rows of ${V^{\prime }}_{p}(t,t)$ in Eq. (1). Pathway activity is denoted as the most significant data pattern which corresponds to the first row of ${V^{\prime }}_{p}(t,t)$ (Eq.(2))as the elements of *S_P _*(*k,t*) are sorted from the highest to the lowest (Additional File 1). The latter rows correspond to the other patterns which significances are determined with the associated eigenvalues. The matrix ${V^{\prime }}_{p}(t,t)$

is orthonormal matrix; therefore the rows represent different data patterns. The two sets of circadian patterns in glycine, serine and threonine metabolism (Figure 7) are retrieved via the first two rows of ${V^{\prime }}_{p}(t,t)$. ${V^{\prime }}_{p}(t,1)$ and ${V^{\prime }}_{p}(t,2)$ have high correlation with fitted sinusoidal model (Additional File 2.). The p-value of ${V^{\prime }}_{p}(t,1)$ is statistically significant whereas the p-value of ${V^{\prime }}_{p}(t,2)$ is not statistically significant.

Table 1 provides the detailed list of identified pathways in each cluster. In total, there are 78 pathways in five clusters. The list of genes in these pathways, associated gene expressions, the weights, the correlation between fitted sinusoidal model and the individual gene expressions can be found in Additional File 3. The identification of the circadian signatures at the pathway level identified biologically relevant processes. As such, gene expression, metabolite concentration and enzyme activity in energy metabolism (e.g. glycolysis and gluconeogenesis), amino acid metabolism (e.g. lysine degradation, urea cycle) [23,24], lipid metabolism (e.g. fatty acid biosynthesis) [25] and DNA replication and protein synthesis (e.g. DNA replication reactome, Purine metabolism) [26] exhibited having circadian dynamics in mammals liver.

**Table 1:** Circadian pathways and associated cluster numbers.

Pathway name	Cluster ID
**ASCORBATE AND ALDARATE METABOLISM**	1

**BUTANOATE METABOLISM**	1

**PURINE METABOLISM**	1

**LIMONENE AND PINENE DEGRADATION**	1

**DNA POLYMERASE**	1

**ATP SYNTHESIS**	1

**DNA REPLICATION REACTOME**	1

**LYSINE DEGRADATION**	1

**HISTIDINE METABOLISM**	1

**PHENYLALANINE METABOLISM**	1

**3 CHLOROACRYLIC ACID DEGRADATION**	1

**G1 TO S CELL CYCLE REACTOME**	2

**FATTY ACID METABOLISM**	2

**BILE ACID BIOSYNTHESIS**	2

**UREA CYCLE AND METABOLISM OF AMINO GROUPS**	2

**VALINE LEUCINE AND ISOLEUCINE DEGRADATION**	2

**TRYPTOPHAN METABOLISM**	2

**P53 SIGNALING PATHWAY**	2

**CELL CYCLE KEGG**	2

**G2 PATHWAY**	2

**ARGININE AND PROLINE METABOLISM**	2

**RNA POLYMERASE**	2

**IFNA PATHWAY**	2

**ST TYPE I INTERFERON PATHWAY**	2

**POLYUNSATURATED FATTY ACID BIOSYNTHESIS**	3

**CELL COMMUNICATION**	3

**ANTIGEN PROCESSING AND PRESENTATION**	3

**MRP PATHWAY**	3

**FRUCTOSE AND MANNOSE METABOLISM**	3

**TYROSINE METABOLISM**	3

**ETC PATHWAY**	4

**TYROSINE METABOLISM**	4

**MALATEX PATHWAY**	4

**PROTEASOME PATHWAY**	4

**ALANINE AND ASPARTATE METABOLISM**	4

**GLYCOLYSIS AND GLUCONEOGENESIS**	4

**SA CASPASE CASCADE**	4

**CHOLESTEROL BIOSYNTHESIS**	5

**GLYCEROPHOSPHOLIPID METABOLISM**	5

**TERPENOID BIOSYNTHESIS**	5

**RNA TRANSCRIPTION REACTOME**	5

**BIOSYNTHESIS OF STEROIDS**	5

**CIRCADIAN EXERCISE**	5

**CYANOAMINO ACID METABOLISM**	5

**FEEDER PATHWAY**	5

**GLYCEROLIPID METABOLISM**	5

**GLYCINE SERINE AND THREONINE METABOLISM**	5

**METHIONINE METABOLISM**	5

**LYSINE BIOSYNTHESIS**	5

**NUCLEOTIDE SUGARS METABOLISM**	5

**ETHER LIPID METABOLISM**	5

**SPHINGOLIPID METABOLISM**	5

**ONE CARBON POOL BY FOLATE**	5

**BASAL TRANSCRIPTION FACTORS**	5

**CIRCADIAN RHYTHM**	5

**LYSINE BIOSYNTHESIS**	5

**LYSINE DEGRADATION**	5

	

**MEF2 D PATHWAY**	5

**METHANE METABOLISM**	5

**METHIONINE METABOLISM**	5

**METHIONINE PATHWAY**	5

**ONE CARBON POOL BY FOLATE**	5

**SA G1 AND S PHASES**	5

**SELENOAMINO ACID METABOLISM**	5

**TID PATHWAY**	5

**TOLL PATHWAY**	5

**APOPTOSIS**	5

**APOPTOSIS GENMAPP**	5

**CARM ER PATHWAY**	5

**EPONFKB PATHWAY**	5

**FXR PATHWAY**	5

**G1 PATHWAY**	5

**GSK3 PATHWAY**	5

**LEPTIN PATHWAY**	5

**P53 PATHWAY**	5

**RACCYCD PATHWAY**	5

**SA REG CASCADE OF CYCLIN EXPR**	5

**TALL1 PATHWAY**	5

*) Since gene products can function in multiple pathways, some pathways that may not be active in liver can be identified as circadian. For example small cell lung cancer, SNARE interactions in vesicular transport, prion disease are not defined in liver tissue. For the statistical analysis, we are not biased by the tissue specific pathways; however an additional filtering is performed for the biologically relevant pathways.

In addition, we evaluated the enrichment of the pathways with the genes that exhibited circadian patterns in [9]. MSigDB database [18] offers an annotation tool that explore gene set annotations to gain further insight into the biology behind a gene set in question. The end result is a p-value indicating the significance of the overlap of the genes with a pathway http://www.broadinstitute.org/gsea/msigdb/annotate.jsp.

The genes that exhibit circadian dynamics in [9] have been mapped to 34 pathways (Additional File 4), nine of which have significant p-value < 0.05.

**Figure 8:**
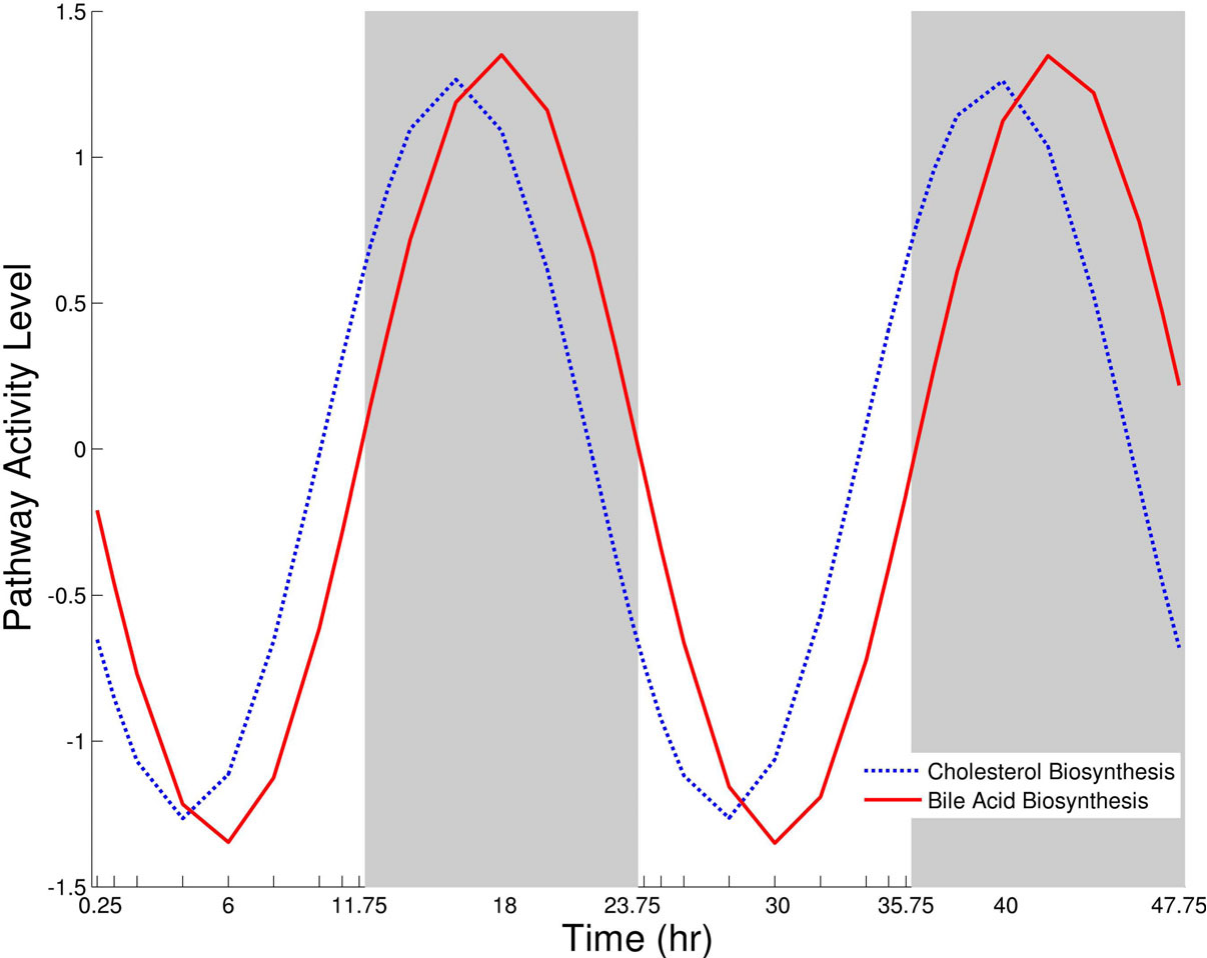
**Fitted sinusoidal models of pathway activity levels for cholesterol biosynthesis and bile acid biosynthesis**.

To further explain the biological significance of the pathway activity level analysis, we studied the coordination between different pathways that is another level of organization in cellular processes, especially in cases where the product of one pathway is the substrate of another pathway. One classic example is the production of bile acids and it needs cholesterol as its starting material. Previous studies have shown that the pathways for steroid and bile acid biosynthesis are coordinated and coupled with cholesterol biosynthesis pathway for maximizing the efficiency of these processes. It has been established that bile acid levels are tightly controlled to ensure appropriate cholesterol catabolism, and promote optimal solubilization and absorption of fat and other essential nutrients [25,27]. Figure 8 shows the fitted sinusoidal models of PAL curves for cholesterol and bile acids biosynthesis. From the Figure 8, we could see that both pathways shows circadian rhythmicity with the phase of oscillations for cholesterol biosynthesis with a peak reaching at 15 hours after lights on, but the bile acid biosynthesis pathway shows a slight time lag in its oscillation with the peak occurring at 17 hours after lights on. In the figure, the PAL curves reach its peak during the mid-dark period and nadir during the mid-light period. As mentioned previously, the peak and nadir of PAL curves represent the maximum variation in the temporal gene expression in the pathway and the exact reverse of the PAL curve is mathematically same as the PAL curve itself (PAL-PAL). But from the literature, we know that these pathways peak during the dark period when the animals are actively feeding. Furthermore, the circadian oscillations in expression of many of the genes involved in the pathway (including the rate limiting genes like HMGCR for cholesterol biosynthesis [16] and CYP7A1 for bile acid biosynthesis [28] peaks during the dark/active period in the 24 hours light/dark cycle. So to deduce the biological significance of the PAL curve, along with the PAL curve pattern one should take into account of the oscillation patterns of the individual gene expression (including the rate limiting genes) along with any existing knowledge about the biological function and regulation of a given pathway. Additional file 5 and 6 provides the expression of individual genes in these pathways. Similar coupling of pathways are observed such as folate biosynthesis and one carbon pool by folate are coupled with purine and pyrimidine metabolism [29].

## Discussion

The goal of this study is to characterize the dynamic evaluation of pathways based on transcriptional profiling. Pathway activity level formulation enabled us to identify circadian signatures of pathways by reducing the overall gene expression level to a single response. We improved the former formulation of the pathway activity level analysis with an additional significance analysis that enhanced our ability to detect relevant circadian changes and reduce the false positives.

Synthetic data was used to demonstrate that pathway activity levels formulation are more robust than the individual gene expression levels in identifying underlying circadian expression pattern within a pathway. It was shown that pathway activity levels can capture the orchestrated change of all the gene expression within a pathway, whereas analysis at the individual gene expression levels could miss moderate but steady changes in the gene expression levels within a pathway. In addition, synthetic data is used to illustrate that the significance analysis of pathway activity levels is necessary to evaluate whether the identified circadian pattern is significant. Even though pathway activity levels identify a circadian pattern, the data captured by the pathway activity levels may not be significant and can be associated with random variations in the data.

In addition, we evaluated pathway activity levels based on a rich time series of transcriptional profiling in rat liver [9] where the rats were maintained in 12:12 light/dark cycle and exposed to the least possible environmental disturbances to minimize stress. Unlike the synthetic data, we did not know the underlying patterns in the microarray data. As a result of the clustering analysis, the most populated patterns of pathway activity levels exhibited circadian rhythms (Figure 3). The over-representation of specific patterns in the data cannot be explained by random events. Therefore, we can conclude that pathway activity level can identify the underlying circadian pattern in the data.

The five main clusters shown in Figure 3 represent the presented curves and the exact reverse curves; PAL = (-) PAL. The turning points can characterize both the peak and the nadir points in biochemical processes. In Figure 3, the signs of PALs are chosen so that PALs have the similar patterns for a better representation and clustering purposes. The sign of PAL can be chosen based on the pattern the genes that have the highest contribution to PAL. For example, we represent pathway activity levels of cholesterol biosynthesis and bile acid synthesis peaking in dark period (Figure 8). From the literature; we know that these pathways peak during the dark period when the animals are actively feeding.

Moreover, the list of the genes that exhibit circadian dynamics were mapped to 34 pathways. Our unsupervised approach identified the entire 34 mapped pathway, whereas nine of mapped pathway exhibited statistically significant enrichment. Additional biologically relevant pathways were identified by pathway activity level analysis such as pathways related to cell cycle, DNA replication and apoptosis exhibited having circadian dynamics in mammals [26,30]. Similar to synthetic data, analysis of biological data emphasizes studying at the individual gene expression levels could miss changes at the pathway level.

Characterizing the circadian regulation at the pathway level is an important piece of information that may help reveal the complex relationships such as understanding the liver functioning. The biological relevance of pathway activity level formulation to analyze circadian rhythms is well illustrated by analyzing coupled pathways. As shown in Figure 8, PAL analysis suggests that bile acid biosynthesis pathways are intrinsically coupled with cholesterol biosynthesis pathway, which is the case as reported by previous studies. Furthermore, this is physiologically important as cholesterol is an important substrate for the biosynthesis of both bile acids. Bile acids are involved in the digestion of dietary lipids and higher levels of bile acid biosynthesis occur during the dark period which represents the active feeds period in rats.

Moreover, we observe series of pathways related to protein synthesis and degradation having circadian patterns. Studies examining the gene expression and enzyme activities related to amino acid metabolism showed persistent circadian rhythms [17]. These studies indicate that amino acid metabolism components tend to correlate with food intake. Though no conclusive evidence is available, transport and metabolic substrates of amino acids have shown clock-regulated changes.

This current analysis is limited, as any pathway method, by currently available pathway knowledge. For example, there are two genes, SHMT1 and SHMT2, which have exactly opposite circadian oscillations in gene expression and hence opposite weights. SHMT1 is a cytosolic enzyme and SHMT2 is a mitochondrial enzyme. Though they catalyze the same reaction, the cellular purposes of these enzymes are different. In addition, several genes not linked to known pathways are not considered in pathway analysis. As more specific pathway databases such as tissue specific pathway databases or cellular compartment specific pathway databases are created and the pathway knowledge databases are improved, the power of this pathway analysis method will increase. Another limitation of this study is that it looks the dynamics of the pathway only at the mRNA levels. But it is a known fact that many biological processes are also regulated at the levels of translation of proteins (like microRNA regulation), activation state (phosphorylation, functionalization, etc), degradation and interaction with other proteins. But again this is just the limitation of the dataset available and we are confident that the methodology can be applied to any proteomics, microRNA arrays dataset, etc in the same way as we applied for our dataset.

## Conclusions

In summary, rather than assessing the importance of a single gene beforehand and map these genes onto pathways, we instead examined the orchestrated change within a pathway. Pathway activity level analysis could reveal the underlying circadian dynamics in the microarray data with an unsupervised approach and biologically relevant results were obtained. We believe that our analysis of circadian pathways based on transcriptional profiling can contribute to filling the gaps between circadian regulation and biochemical activity. While transcriptional profiling is a valuable tool for unrevealing potential connections between the circadian clock and biochemical activity [31], complementing the transcriptional studies with proteomic and metabolomics analyses will provide new insights to the circadian phenomenon.

## Authors' contributions

MAO and SS performed the analysis. RRA, DCD and WJJ assisted in data interpretation. IPA supervised the study. All authors read and approved the final manuscript.

## Supplementary Material

Additional file 1 **The relative values of the associated eigenvalues for glycine, serine and threonine metabolism**. The bars indicate the variation in the data captured by each individual eigenvector for glycine, serine and threonine metabolism pathways. T solid line represents the data variability captured by the corresponding eigenvectors when randomly generated data (of the same dimension) were used. No apparent distinction between the actual data and randomly generated data was identified after the first eigenvalue, as quantified by the calculated *p*-values. Click here for file

Additional file 2 The first 4 rows of ${V^{\prime }}_{p}(t,t),$ that are retrieved from SVD calculations of Glycine, serine and threonine metabolism the elements of *S_P _*(*k*, *t*) are sorted from the highest to the lowest. 1)${V^{\prime }}_{p}(t,1),$ 2) ${V^{\prime }}_{p}(t,2),$ 3) ${V^{\prime }}_{p}(t,3),$ 4) ${V^{\prime }}_{p}(t,1),$ Click here for file

Additional file 3 **Pathway activity levels of five clusters and associated information of the genes in pathways**. The excel file contains two sheets. First sheet, Pathway Activities includes the pathway activity levels and associated cluster numbers. Second Sheet contains the genes in selected pathways and associated information such as gene expression, weights and correlations. Click here for file

Additional file 4 **Enriched pathways by circadian genes**. The circadian genes were mapped to canonical pathways provided by http://www.broadinstitute.org/gsea/msigdb/. p-values indicate the significance of the overlap of the circadian genes within a pathway Click here for file

Additional file 5 **Individual gene expressions in cholesterol biosynthesis**. Associated weights and correlations with the fitted sinusoidal model were given on top of each panel. Click here for file

Additional file 6 **Individual gene expressions in bile acid biosynthesis**. Associated weights and correlations with the fitted sinusoidal model were given on top of each panel. Click here for file
