# Supplementary figures and images for: Circadian signatures in rat liver: from gene expression to pathways
Source: BMC Bioinformatics. 2010 Nov 1;11:540. doi: 10.1186/1471-2105-11-540 (PMC2990769; doi:10.1186/1471-2105-11-540)

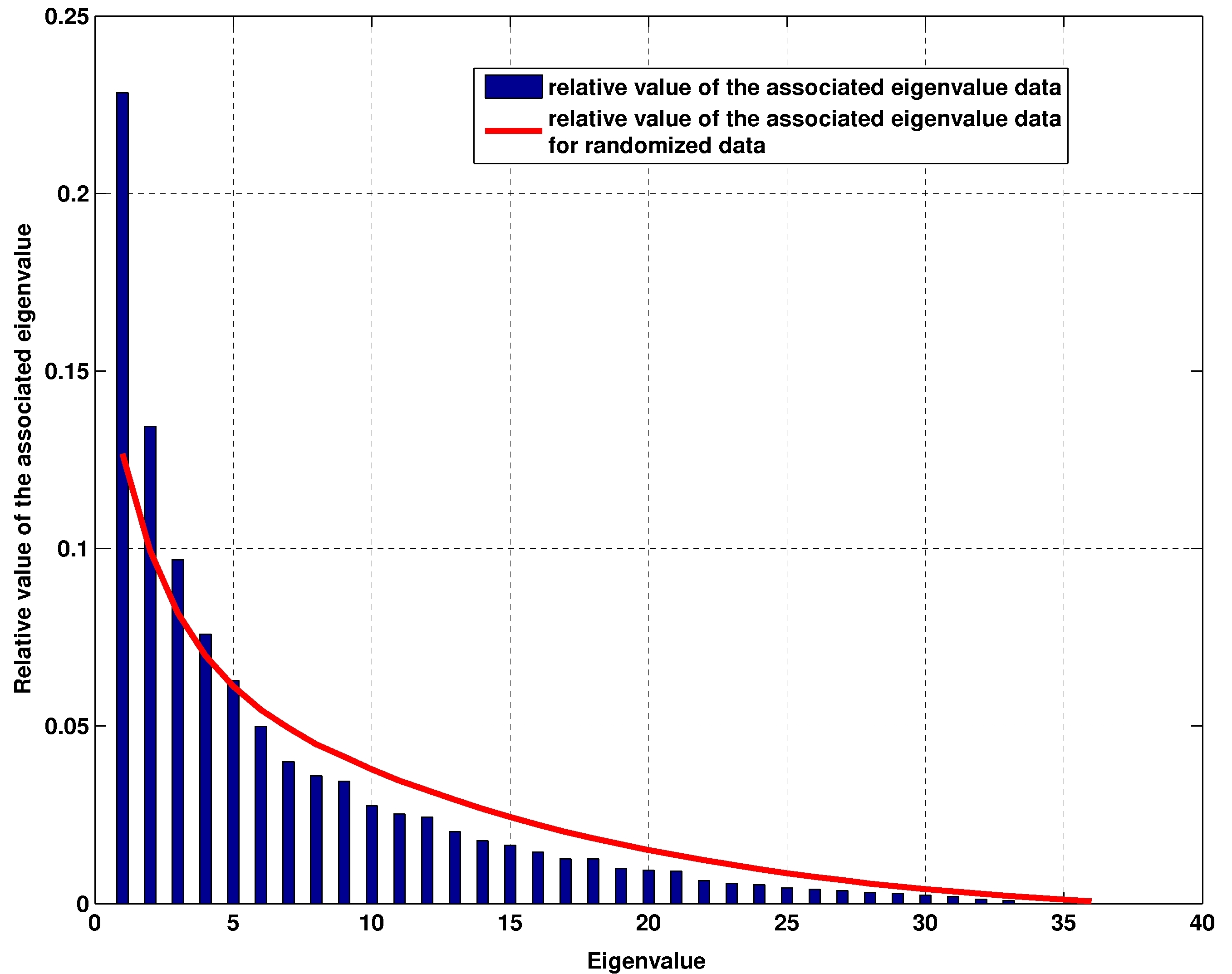

Supplement: Additional file 1 — The relative values of the associated eigenvalues for glycine, serine and threonine metabolism. The bars indicate the variation in the data captured by each individual eigenvector for glycine, serine and threonine metabolism pathways. T solid line represents the data variability captured by the corresponding eigenvectors when randomly generated data (of the same dimension) were used. No apparent distinction between the actual data and randomly generated data was identified after the first eigenvalue, as quantified by the calculated p-values. [file 1471-2105-11-540-S1.JPEG]

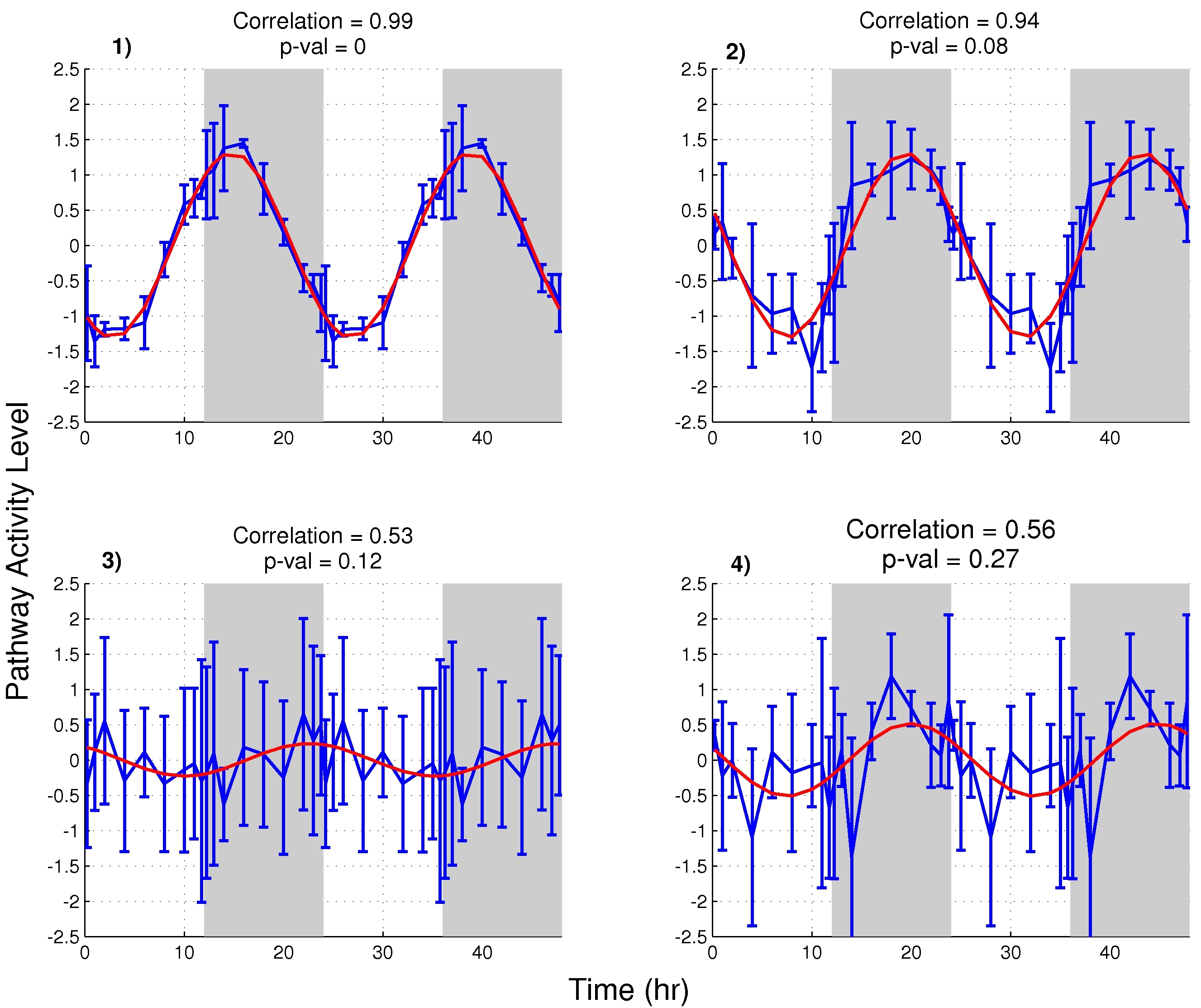

Supplement: Additional file 2 — The first 4 rows of V′p(t,t), that are retrieved from SVD calculations of Glycine, serine and threonine metabolism the elements of SP (k, t) are sorted from the highest to the lowest. 1)V′p(t,1), 2) V′p(t,2), 3) V′p(t,3), 4) V′p(t,1), [file 1471-2105-11-540-S2.JPEG]

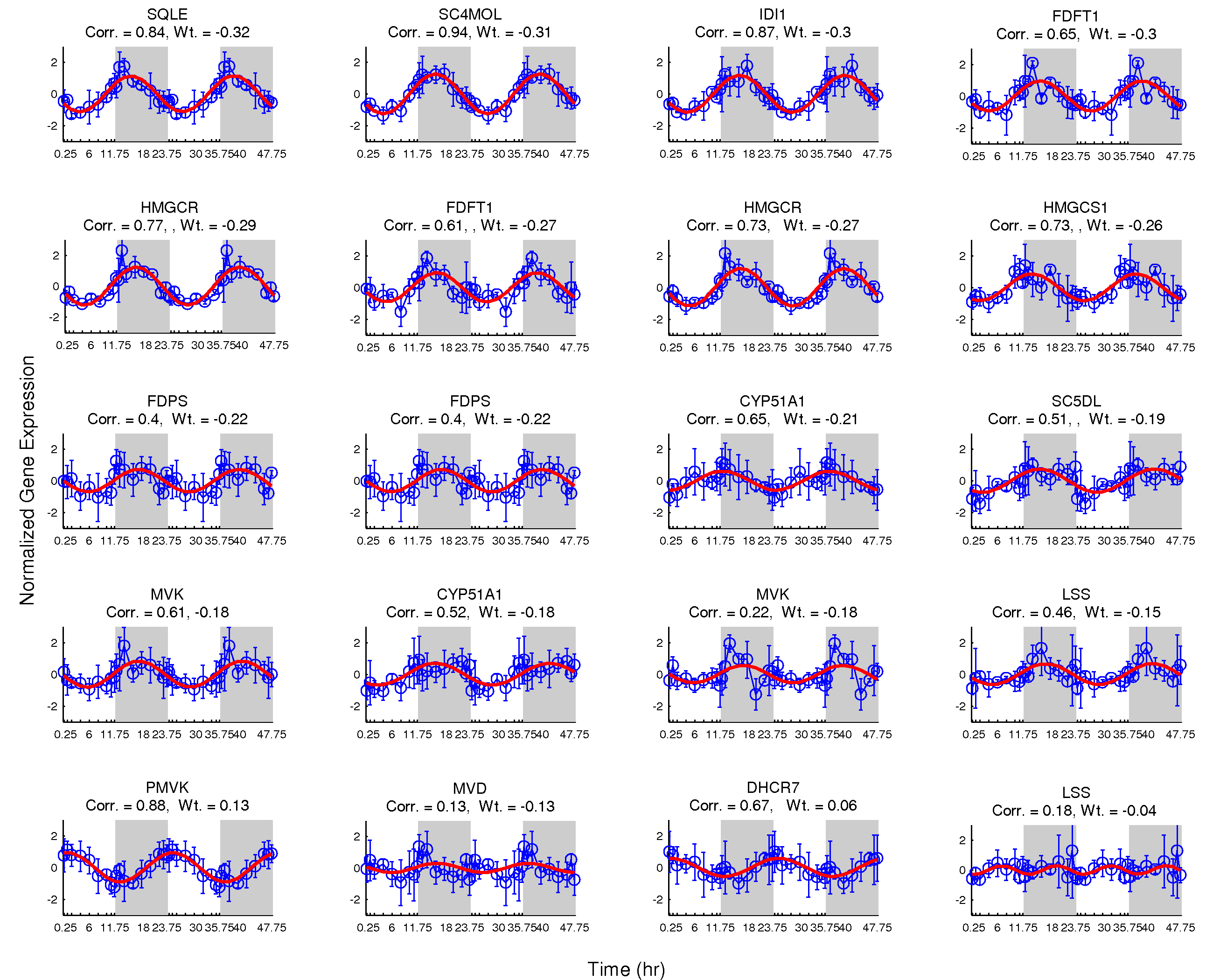

Supplement: Additional file 5 — Individual gene expressions in cholesterol biosynthesis. Associated weights and correlations with the fitted sinusoidal model were given on top of each panel. [file 1471-2105-11-540-S5.PNG]

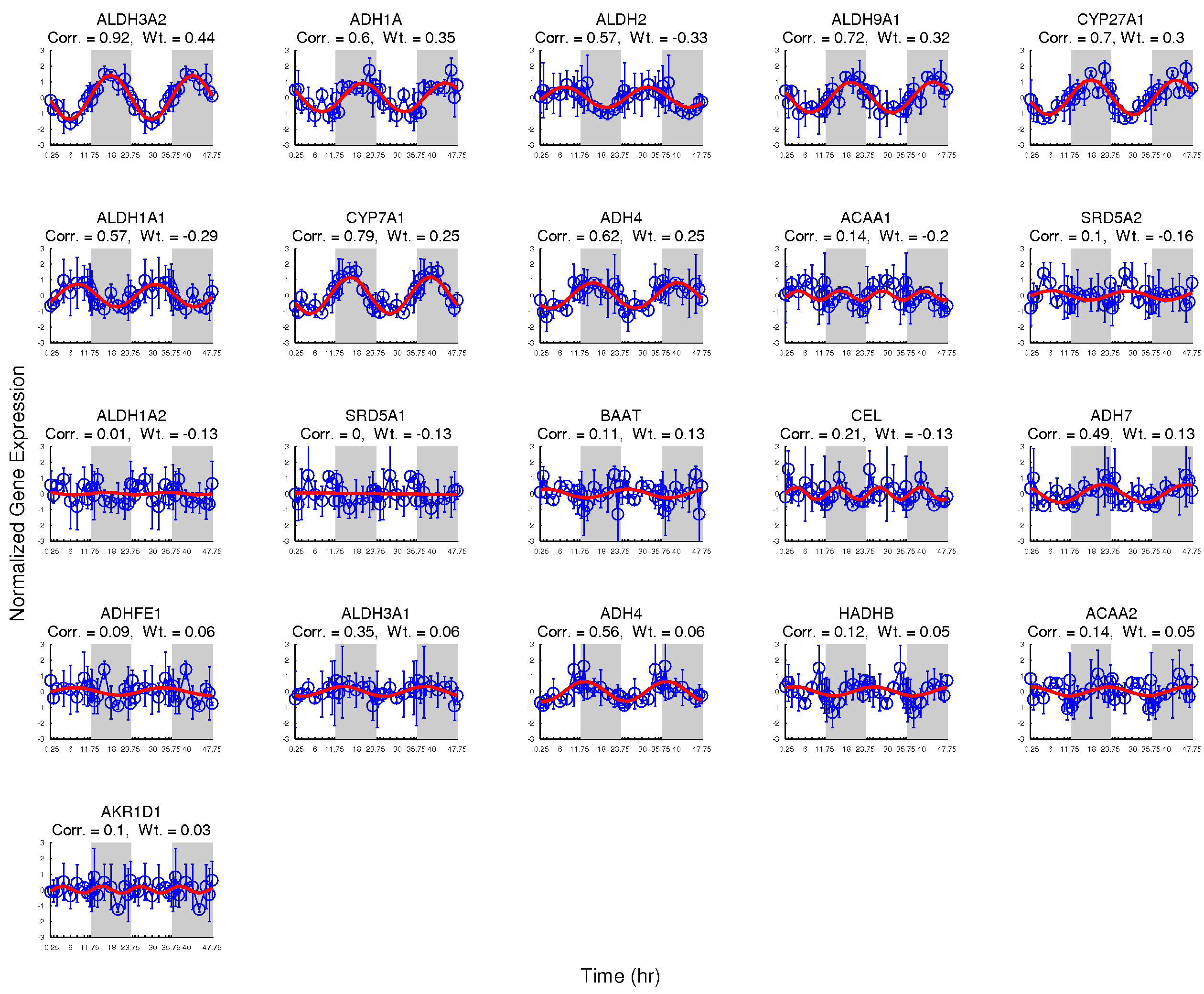

Supplement: Additional file 6 — Individual gene expressions in bile acid biosynthesis. Associated weights and correlations with the fitted sinusoidal model were given on top of each panel. [file 1471-2105-11-540-S6.PNG]
